# Mainstreaming adult ADHD into primary care in the UK: guidance, practice, and best practice recommendations

**DOI:** 10.1186/s12888-022-04290-7

**Published:** 2022-10-11

**Authors:** Philip Asherson, Laurence Leaver, Marios Adamou, Muhammad Arif, Gemma Askey, Margi Butler, Sally Cubbin, Tamsin Newlove-Delgado, James Kustow, Jonathan Lanham-Cook, James Findlay, Judith Maxwell, Peter Mason, Helen Read, Kobus van Rensburg, Ulrich Müller-Sedgwick, Jane Sedgwick-Müller, Caroline Skirrow

**Affiliations:** 1 Social Genetic and Developmental Psychiatry, Institute of Psychiatry, Psychology and Neuroscience, Kings College London, De Crespigny Park, London, SE5 8AF UK; 2 Green Templeton College, Oxford, UK; 3 University of Huddersfield, Huddersfield, UK; 4 Leicestershire Partnership NHS Trust, Leicester, UK; 5 NHS Warrington Clinical Commissioning Group, Warrington, UK; 6 Manor Hospital, Oxford, UK; 7 University of Exeter, Exeter, UK; 8 Barnet, Enfield and Haringey Mental Health NHS Trust, London, UK; 9 Warrington Primary Care Community Interest Company, Warrington, UK; 10 NHS Northamptonshire Clinical Commissioning Group, Northampton, UK; 11 Inclusion Health Care, Leicester, UK; 12 ADHD And Psychiatry Services Limited, Liverpool, UK; 13 Oxleas Foundation Trust, London, UK; 14 Northamptonshire Healthcare NHS Foundation Trust, Kettering, UK; 15 Novoic Ltd, London, UK

**Keywords:** Adult, Attention deficit disorder with hyperactivity, Primary health care, Secondary care, Tertiary healthcare, Delivery of healthcare, Delivery of health care, integrated, Continuity of patient care, service delivery, National Institute of health and care excellence (NICE), UK adult ADHD network (UKAAN)

## Abstract

**Background:**

ADHD in adults is a common and debilitating neurodevelopmental mental health condition. Yet, diagnosis, clinical management and monitoring are frequently constrained by scarce resources, low capacity in specialist services and limited awareness or training in both primary and secondary care. As a result, many people with ADHD experience serious barriers in accessing the care they need.

**Methods:**

Professionals across primary, secondary, and tertiary care met to discuss adult ADHD clinical care in the United Kingdom. Discussions identified constraints in service provision, and service delivery models with potential to improve healthcare access and delivery. The group aimed to provide a roadmap for improving access to ADHD treatment, identifying avenues for improving provision under current constraints, and innovating provision in the longer-term. National Institute for Health and Care Excellence (NICE) guidelines were used as a benchmark in discussions.

**Results:**

The group identified three interrelated constraints. First, inconsistent interpretation of what constitutes a ‘specialist’ in the context of delivering ADHD care. Second, restriction of service delivery to limited capacity secondary or tertiary care services. Third, financial limitations or conflicts which reduce capacity and render transfer of care between healthcare sectors difficult. The group recommended the development of ADHD specialism within primary care, along with the transfer of routine and straightforward treatment monitoring to primary care services. Longer term, ADHD care pathways should be brought into line with those for other common mental health disorders, including treatment initiation by appropriately qualified clinicians in primary care, and referral to secondary mental health or tertiary services for more complex cases. Long-term plans in the NHS for more joined up and flexible provision, using a primary care network approach, could invest in developing shared ADHD specialist resources.

**Conclusions:**

The relegation of adult ADHD diagnosis, treatment and monitoring to specialist tertiary and secondary services is at odds with its high prevalence and chronic course. To enable the cost-effective and at-scale access to ADHD treatment that is needed, general adult mental health and primary care must be empowered to play a key role in the delivery of quality services for adults with ADHD.

## Background

ADHD is a neurodevelopmental disorder that develops during childhood or early adolescence and frequently persists into adulthood, where it is then referred to as adult ADHD. The disorder is defined by a persistent pattern of inattention and/or hyperactivity-impulsivity that interferes with or reduces the quality of functioning in daily life [1]. ADHD first presents in childhood with onset of several symptoms by age 12 and is estimated to affect between 5 and 7% of children and adolescents worldwide [2, 3], and between 2.5–3.4% of adults [4, 5].

Follow-up studies of children with ADHD show that symptoms and impairment frequently persist into adulthood. An initial review of follow-up studies, mainly from the United States, estimated that 15% of children with ADHD retained the full diagnosis by the age of 25 years [6]. Further studies estimated higher persistence rates (50–80%) across different countries [7–9]. In these studies the higher rates are potentially driven by the greater severity of symptomatology in childhood and the greater reliance on informant data when establishing the diagnosis at follow-up [8, 10, 11]. A recent review of 20 nationally or regionally representative world mental health surveys using the Composite International Diagnostic Interview administered to 26,744 adults found prevalence of DSM-IV ADHD was 2.8% across surveys [12]. This study demonstrated that adult ADHD is a common, impairing, and highly comorbid condition, but vastly under-recognized and undertreated across countries and cultures.

Adverse outcomes of ADHD are well-documented and often severe. These include social and relationship problems [13], delinquency [14], involvement with the criminal justice system [15], substance abuse [16], increased rates of communicable diseases [17], accidents, injuries, and mortality [18, 19], problems at school and educational underachievement [20, 21], under-employment and occupational problems [20, 22], and increased rates of homelessness [23]. A further source of impairment is the high rate of co-existing mental health disorders in people with ADHD [12], reported to be as high as 90% [24]. These include specific learning difficulties (dyslexia and dyspraxia), anxiety disorders, depression, bipolar disorder, personality disorder, substance abuse disorders, and other neurodevelopmental disorders such as autism [25–28].

The benefits of treatment are well documented and can reduce both immediate and long-term risks and adverse outcomes [29, 30]. Delays in effective treatment are likely to reduce economic productivity for the individual, and increase public costs including healthcare, social care and payment of state benefits [31, 32]. Pharmacoepidemiological studies provide an important source of evidence for long-term benefits of treating ADHD [30], and demonstrate a wide range of societal benefits, including reduced rates of criminality [33] and violent aggression [34]. Evidence suggests broader health-related benefits of engaging with treatment for ADHD, including lower risks of serious transport accidents [35], depression [36], suicidality [37], and substance use disorders [38], and improved seizure control in patients with ADHD and epilepsy [39]. Pharmacotherapy is also associated with decreased risk of communicable disease contraction, including influenza [40] and COVID-19 [41] for patients with ADHD. Although suggestive of a pharmacological effect, these studies do not confirm a causal relationship with ADHD medication but do imply broad benefits of engaging with the treatment process.

National and consensus guidelines for the treatment of ADHD in adults are similar across the world [42–46], including the guidance from the National Institute for Health and Care Excellence (NICE) in England and Wales [47, 48]. These indicate that ADHD causes significant impairment across the lifespan and that efforts need to be made to recognize and treat ADHD in adults to reduce impact on function and mental health in daily life.

Like other guidelines for adult ADHD in European countries and worldwide, NICE recommend that diagnostic assessments should be conducted by adult mental health care professionals with training in diagnosis and treatment of ADHD. Thus, NICE guidelines recommend a higher standard of expertise for assessment and treatment than for other common mental health disorders such as anxiety and depression, which are frequently managed solely by non-specialists within primary care. In the UK and elsewhere, ADHD is mainly managed by tertiary and some secondary mental health services with relevant expertise. Whilst such specialization ensures appropriate targeting of treatments for adult ADHD, over-specialization runs the risk of limiting access to diagnostic assessments and treatment.

Treatment rates for ADHD are far lower than expected. Raman et al. (2018) used population-based databases from 13 countries between 2001 and 2015, and applied a common approach to define study populations and parameters across countries [49]. Rates of treatment increased over the period studied. However, rates of medication use for ADHD in adults stood at only 0.05% of the UK population and 0.39% pooled prevalence across countries, far below rates of community prevalence. Expected treatment rates should be in the order of 1% or more of adults, even if we only focus on the most severe cases. Overall, figures therefore indicate a considerable unmet need in the diagnosis and treatment of ADHD in adults. This is reflected in national surveys in the UK, showing that services for ADHD are uncommon, under-resourced and ‘patchy’ [50–52], and a range of reports show extremely long waiting lists for adults seeking assessment and treatment [53–58]. International colleagues confirm that difficulties with service delivery for adult ADHD are widespread, thus potential solutions to this situation are of general interest.

In the context of guidance from NICE [48], evidence for the cost-effectiveness of diagnosing and treating adult ADHD and the presence of service delivery gaps, there is clearly room for improvement in the management of adult ADHD. One approach has been a move towards including ADHD as a core competency in generic secondary care adult mental health services. Adult ADHD can be a severe condition with a high burden of comorbidity, with impairments that are similar to those currently managed in secondary care. There is evidence that a substantial proportion (17–22%) of patients treated by secondary care mental health services have undiagnosed ADHD [59, 60], indicating a need to look below the surface of anxiety, mood and personality disorder symptoms for the presence of ADHD. Adequate detection and treatment of ADHD is likely to improve the management and outcomes of patients who are already on the caseload of adult mental health services.

However, ADHD is not always so complex or severe that it meets the requirements for involvement of secondary or tertiary care mental health services. Integration of ADHD services into more generalist clinical services could therefore also occur, within clear parameters, at the level of primary care, following treatment models for other common mental health problems such as anxiety disorders and depression. It is in this context that we set out to consider the potential role of primary care services in the diagnosis and management of adult ADHD and examine new models of care that have developed in recent years. To achieve this, we brought together healthcare professionals from primary, secondary and tertiary care services in the UK to identify bottlenecks in service provision and examine models of service delivery which could help to shift care for ADHD into primary and secondary healthcare systems, to improve healthcare access and delivery for people with ADHD. While these discussions are focused on services in the UK, the same principles are broadly applicable across many countries and regions of the world.

## Methods

A discussion group convened at the Royal College of Physicians in London on the 14th of June 2019. The meeting brought together primary, secondary, and tertiary healthcare practitioners with extensive experience in the clinical management of ADHD in adults, with the primary goal of evaluating the current and future potential role of primary care in the management of adults with ADHD. This was achieved by bringing together different perspectives from professionals across these healthcare sectors and identifying potential solutions for cost-effective delivery of ADHD diagnostic and treatment services. Given that ADHD presents as a common mental health problem with a wide range of severity and different levels of impairment, the discussion examined potential avenues for integrating treatment into primary and secondary care services, while being sensitive to the way in which professionals in each sector work and the pressures they deal with in their day-to-day professional lives.

Attendees discussed the status and integration of ADHD in primary, secondary and tertiary healthcare in the UK, comparing different models of service delivery and their relative success. The group then considered a roadmap for improving access to ADHD treatment, by identifying issues and bottlenecks, and examining how best to bring about change in the short-term, and innovative ADHD provision in the longer-term. Discussions and recommendations were focused on adult ADHD provision in England, whilst recognising that provision and funding models vary across different regions and countries. NICE guidelines were used as a benchmark for service provision, since these provide official guidance for clinical best practice in England [48].

Since the NICE guidelines recommend medication alongside psychoeducation as the first line treatment for ADHD, we focus in this paper on the delivery of services for the diagnostic assessment and initiating and monitoring of medication. The group recommended that psychoeducation, including the teaching of coping skills for ADHD symptoms and impairments, should be integrated into the assessment and follow-up process. Psychological treatments may also be needed, particularly for those with comorbidities, ADHD symptoms and impairments that do not respond to medication, or for individuals who do not wish to take medication.

Meeting attendees included clinicians from primary care (three General Practitioners (GPs) and one nurse consultant); two health commissioning representatives; and professionals specialising in ADHD across a range of mental health professions in secondary or tertiary care (nursing, psychiatry, psychology, public health medicine). One attendee worked primarily in research, and another jointly in research and tertiary care.

The initial debate from the morning session was recorded and transcribed. The transcription from the morning session was synthesised jointly by the lead author (PA) and writer (CS) and circulated to the consensus group and other members of the UKAAN executive committee (JK, JS, MA). In the current document, where relevant and available, consensus discussion points are provided with reference to the supporting research literature, grey literature, policy, or legislative documentation. Draft versions of this report were reviewed by co-authors and the final draft approved by all authors prior to submission. The presented outcomes represent the consensus views of the whole group.

## Results and consensus outcome

### Historical context

In the early to mid-1990s in the UK, ADHD was treated in children through specialist-only tertiary clinics, and pharmacological treatment was relatively rare. Between 1995 and 2005 rates of diagnosis and prescribing for childhood ADHD increased significantly in the UK [61, 62]. These increases occurred alongside a move of ADHD provision from specialist clinics to the mainstream generic child and adolescent mental health services (CAMHS). Even so, there is still evidence for a continued shortfall between diagnosis and treatment rates currently, and the overall prevalence of childhood ADHD in the general population [63].

Service provision for adult ADHD within the UK National Health Service (NHS) was initiated later than that for children. The reasons for this are not fully understood, but for a long time the validity of the diagnosis of ADHD was questioned (see NICE 2008, Chapter 5 [47]), and ADHD was thought to be a disorder that most people grew out of by the adult years [64]. In the mid-90s, services for adult ADHD were restricted to a handful of specialists including clinics in Bristol (Professor David Nutt, addiction psychiatry specialist), Cambridge (Dr Jonathan Dowson, specialist in personality disorder), and London (Professors Brian Toone and Suzy Young, a specialist in neuropsychiatry and a clinical psychologist, respectively). Additional specialist services for adult ADHD were then developed, including clinics led by Dr. Kobus van Rensburg in Northampton, Dr. Muhammad Arif in Leicester, and Professor Marios Adamou in Yorkshire. These early services have continued to develop and have seen year-on-year increases in the number of referrals right up to the current day. However, they only provided services for a very small proportion of adults with ADHD, and most regions of the UK had no available services.

The publication of the NICE Clinical guideline CG72 in 2008 [47] was a landmark in the development of services for adult ADHD. Chapter 5 of the 2008 NICE guidelines addressed the question of the validity of the diagnostic construct in both children and adults, which is the first and only time that NICE have conducted such a review for a clinical condition, due at the time to continued uncertainty within child and adolescent services, and in adult psychiatry in particular [47]. These guidelines clarified that ADHD frequently persists into adulthood, that treatment effects are like those seen in children, and that access to diagnostic and treatment services is required, cost-effective, and should be available throughout England and Wales.

### Adult ADHD clinical provision in the UK

The publication of the 2008 NICE guidelines led to a rapid expansion in the number of clinics across England and Wales after 2008, and particularly in the last decade. Many regions set up specialist tertiary clinics for ADHD or neurodevelopmental disorders (ADHD plus autism) [48], although some regions lacked any services for ADHD. As with the early service models for children, adult ADHD diagnosis and treatment was restricted to specialist tertiary services. However, in more recent years an increasing number of ADHD services have been integrated into generic secondary adult mental health care, and in a few cases even into primary care. The ‘CATCh-uS’ mapping study which aimed to identify all adult ADHD services in England and Wales, listed over 40 NHS specialist services for adult ADHD and around 100 NHS adult services supporting adults with ADHD in community mental health teams and adult learning disability practices [51, 52, 65]. Mirroring the integration of childhood ADHD into general child and adolescent mental health services, we envisage that in the future most, if not all, adult psychiatrists will diagnose and treat ADHD as part of their general approach to adult mental health.

With increased recognition of ADHD, and improvements in service availability and provision, there has been a rapid increase in prescribing medication for ADHD [61, 66]. However, prescription rates remain lower than expected. A large population survey completed in 2014 identified around one in ten adults in the UK as having sufficient ADHD characteristics to warrant a clinical assessment for ADHD, but only a small proportion of those screening positive had ever been diagnosed with ADHD (2.3%), and even fewer (0.5%) were currently taking medications indicated for ADHD [50]. The low rate of medical treatment for adult ADHD worldwide is well documented [49].

In children and adolescents there is also a discrepancy between the rates of ADHD treatment (0.2–0.9% since the mid-2000s [63]) and the estimated prevalence of around 5% [2, 3]. Inevitably, a proportion of children with undiagnosed ADHD present for ADHD assessment and treatment for the first time in adulthood. Furthermore, there is increasing evidence that in a subset, ADHD emerges as a clinically impairing condition between the ages of 12–17 [67]. One survey of 89 adults with ADHD in the UK showed that just under half of respondents (45%) were diagnosed for the first time in adulthood [13]. Similar results were reported in a large pan-European survey of adults with self-reported ADHD, where just over half of participants (52%) received a diagnosis of ADHD for the first time in adulthood [68].

Scant provision for ADHD is also reflected in national surveys. A National Health Service (NHS) survey of health and wellbeing in 2014 described mental health services for adult ADHD as relatively uncommon or greatly under-resourced [50]. The CATCh-uS mapping study described adult ADHD service provision as ‘patchy’ [51, 52]. Only 12 out of 294 services provided the full range of treatments recommended by NICE, and there was considerable geographical variation in the availability of services. Related analysis of primary care also found significant regional variations in prescribing for ADHD and referral rates to adult mental health services [69].

A 2018 audit by Takeda pharmaceuticals used data from Freedom of Information requests to survey ADHD provision in Clinical Commissioning Groups (CCGs: regional NHS bodies that allocate, plan, and provide services for populations within specific service regions). They identified considerable regional variation in waiting times for adult ADHD assessment, from as short as 4 weeks to as long as 3.8 years [53]. Substantial regional variations in service delivery, and extremely long waiting times for adults seeking assessment and treatment have been highlighted in local and national media reports [55–58]. Overall, these indicate a substantial unmet need for adults with ADHD.

### ADHD and transition to adult services

Many children diagnosed with ADHD continue to meet diagnostic criteria into adulthood [6, 70], and require continued support and treatment for their symptoms as they transition into adult care. However, ADHD medication prescribing declines rapidly during teenage years, with sharp decreases in prescribing co-occurring with transition from child to adult services and simultaneous increases in the prescribing of other psychotropic medication [71, 72]. This reduction in treatment exceeds that expected from the developmental decline in ADHD symptoms, which alongside other evidence documenting failures in transition [73–78], indicates that older teenagers and young adults are likely to be undertreated or have their treatment or care discontinued prematurely. In the CATCh-uS study, fewer than a quarter of those identified as requiring transition to continue ADHD medication made a ‘successful’ transition, defined as attending a first appointment in an adult service [78].

Risks to maintenance of stable treatment regimens in young people occur when at transition age they must wait for access to congested adult ADHD services, or the required specialist adult services are simply not available in their locality. Young people are more likely to experience difficulties in maintaining treatment regimens where transition is interrupted in parts of the UK where primary care practitioners do not routinely prescribe for children with ADHD but are suddenly expected to take over prescribing when they transfer to adult services. In these cases primary care practitioners may become involved in daily treatment management ‘by default’ [79].

The clinical experts in the group agreed that for many young people who are effectively managed and on stable treatment, transfer of care can be straightforward and not time consuming. However, there will are other cases where treatment is not so straightforward, where significant continued symptoms and impairments, or additional mental health or disabilities, require further assessment and treatment.

### ADHD in higher education

ADHD has a particularly strong negative impact on learning and educational performance, and therefore may present for the first time to student health or disability services. Within student disability services the diagnosis is often treated similarly to specific learning difficulties (SpLD), enabling students to gain access to appropriate educational support. However, providing support for students with ADHD may require a medical diagnosis, especially when the symptoms and impairments are sufficiently severe to warrant treatment with medication.

To address this problem, the SpLD Assessment Standard Committee (SASC) provided guidelines in 2013 and 2021 to improve access to support for students with learning difficulties related to ADHD [80]. SASC recommend that practitioner psychologists and specialist teacher assessors with relevant training can make provisional or (‘non-medical’) diagnoses of ADHD allowing students with ADHD to access support in a timely fashion. Non-medical support for students with specific learning difficulties includes help with the structure, planning and other ‘executive function’ deficits that impact on learning. This can be further enhanced by training in robust coping skills for specific difficulties related to ADHD.

Regarding pharmacological treatment, referral via their GP to specialist services for medical assessment and treatment usually results in students ending up on long waiting lists, often of 2 years or more in the UK. Long waiting lists increase the risk of academic under-performance, educational failure, or comorbid mental health conditions [81]. It was agreed that more rapid access pathways for medical care need to be developed by student primary and secondary mental health services.

### Care pathways

**Fig. 1:**
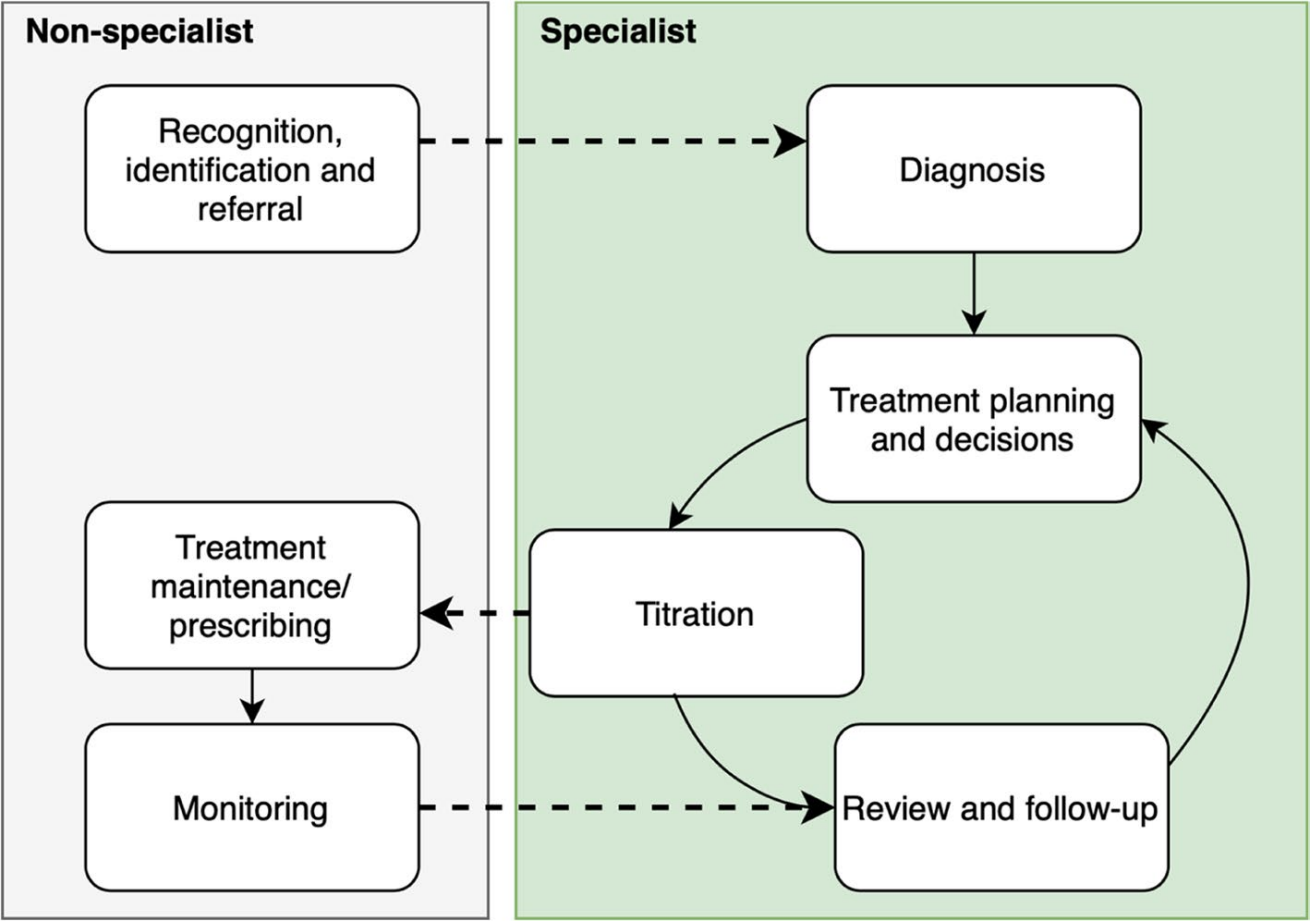
Simplified schema for roles taken by non-specialist (primary care) and specialist healthcare providers in the treatment of ADHD according to NICE guidelines. Dashed lines indicate key areas of communication between general and specialist healthcare providers

NICE guidelines provide a comprehensive approach for assessing and managing ADHD in the NHS in England and Wales, with service organisation centred around multidisciplinary specialist ADHD teams [48]. The guidelines describe assessment and treatment from multidisciplinary specialist teams or clinics, smooth transition from child to adult services for children with ADHD persisting into adulthood, advice on environmental modifications, psychoeducation, and medication for children and adults with moderate or severe symptoms with continuing impairment despite environmental modifications [48]. Once diagnosis has been made, psychoeducation delivered and medication initiated and titrated to a maintenance dose, NICE recommend that routine prescribing and physical monitoring is transferred to primary care through shared care protocols [48]. Once yearly specialist review is then recommended. Figure 1 provides a simplified schema of roles taken by non-specialist and specialist healthcare providers in the treatment pathway for ADHD.

NICE defines an ADHD specialist as a “psychiatrist or paediatrician or other appropriately qualified healthcare professional with training and expertise in the diagnosis of ADHD” [82]. The guidelines state that diagnostic assessment should be completed by “a mental health specialist trained in the diagnosis and treatment of ADHD”, and that “all medication for ADHD should only be initiated by a healthcare professional with training and expertise in diagnosing and managing ADHD” [48]. NICE therefore emphasise the role of ADHD specialists in diagnosis, treatment, and continuity of care. This includes communication with and coordination between primary and secondary/tertiary care, as well as ensuring the availability of age-appropriate psychological services [48]. Specialist ADHD teams are also tasked with developing training programmes for the healthcare workforce, and social care, education and forensic care providers. The function of the specialist ADHD team is therefore multidimensional, seen as a combination of training, facilitation, coordination, consultation, diagnosis, and treatment implementation. The British National Formulary (BNF), which provides national information on selection and clinical use of medicines, similarly recommends ADHD medication initiation under specialist supervision [83, 84]. For children and young people the guidelines specify that diagnosis requires a full assessment completed by a specialist within secondary care [82] and that primary care providers should not diagnose or initiate treatment for ADHD [48]. For young people transitioning into adult care, referral to general adult psychiatric services for assessment is recommended [48]. However, for adults with ADHD the clinical setting of the qualified professional (primary, secondary or tertiary healthcare) is not specified.

### The ADHD ‘specialist’: defining expertise and competencies

The requirement for specialism in the absence of specific guidance of who can gain this specialism and how, allows for a level of flexibility regarding who can become specialist and be relied on as a decision maker for diagnosis and treatment support. However, this flexibility also presents a lack of clarity, making it difficult to ascertain which services are best placed to deliver diagnosis and treatment for ADHD. For example, some psychiatrists take the view that specialist means a subspecialist/tertiary care service, when it could also be considered that the speciality is general adult mental health, to include ADHD. This can lead to difficulties when patients move from one region of the country to another. ADHD diagnosis and treatments formulated by a qualifying specialist in one area may not be recognised as such in another. Lack of clarity surrounding specialism also impacts commissioning services for ADHD, where commissioners can be given conflicting advice from primary, secondary, and tertiary care on the level of specialism and investment required for delivering ADHD services.

It is therefore important to clarify the requirements for specialism in ADHD. Greater consistency in the definition of an ADHD specialist would allow service providers to identify and train clinical staff in the clinical management of ADHD. Furthermore, this could help healthcare services to ratify diagnoses and treatment plans to make these transferrable when patients move between services. It may also help to support healthcare commissioners, such as the current clinical commissioning groups in England, in identifying and funding services that are best placed to deliver health care provision for ADHD.

Mental health professionals from a variety of backgrounds have the foundation of clinical knowledge required to acquire additional specialism in ADHD. These include different categories of professionals (e.g., doctors, psychologists, nurses, pharmacists) from primary, secondary, and tertiary care sectors with appropriate training in adult mental health. Certain groups, such as clinical psychologists and specialists in the assessment of specific learning difficulties may be well placed to acquire additional specialism in diagnosis, and delivery of non-pharmacological interventions. Some groups such as general adult psychiatrists, primary care physicians, and mental health nurses and pharmacists could quite easily acquire the skills required for initiation and ongoing monitoring of pharmacological treatments, once diagnosis has been confirmed. Key competencies for health professionals in diagnosing and treating ADHD in adults are provided in Table 1.

**Table 1:** Recommended competencies required for ADHD specialist in diagnosis, treatment initiation, and medication monitoring, as discussed by the consensus group, based on NICE guideline (2008)

Role	Key competencies
Diagnosis	• Understand normal patterns of development and behaviour. • Differentiate ADHD from normal development and from other mental health disorders (including other neurodevelopmental disorders). • Consider family and social factors. • Evaluate contribution from other medical conditions (e.g., epilepsy). • Evaluate contribution of comorbid mental health conditions. • Consider contextual factors or behaviours which impact on symptoms, impairment, risk, or choice of treatment.
Treatment and monitoring of medication	• Understand pharmacology of medications used in ADHD • Be familiar with widely used preparations: their form, indications, posology, contraindications, special warnings and precautions, interactions (including non-prescription drugs), use in special groups (e.g., pregnancy), adverse effects, pharmacokinetics, risks if used incorrectly, licensing status and costs. • Understand the effect of ADHD medications on comorbid conditions (e.g., mania, psychosis). • Assess for cautions or contraindications for each drug. • Tailor treatment effectively to individual needs (e.g., fine tuning of dose and timing). • Risk assess for drug misuse and diversion. • Monitor and respond to changes in weight, heart rate and blood pressure; how and when to refer to cardiology, or other relevant specialists.
Psychoeducation	• Understanding symptoms and links to impairment in daily life • Understand strategies or coping mechanisms for the management of ADHD symptoms in daily life

Importantly, the management of ADHD is not usually more complex or difficult than other common mental health conditions currently treated within primary and secondary care. In general, those already trained to appropriately diagnose and manage other common mental health disorders, are well placed to acquire the skills required to diagnose and treat ADHD. As things currently stand, however, adult ADHD is typically not incorporated with sufficient detail in generic medical or psychiatric training. Training is therefore required under a mental health professional with clinical expertise of ADHD, or through formal training courses, such as those offered by the UK Adult ADHD Network [85], with appropriate supervision and ongoing peer support.

### Secondary mental health services

Although there has been considerable progress within secondary care mental health services, limited experience and knowledge of ADHD is an ongoing problem. Clinicians working with anxiety, depression, bipolar, personality and other common mental health disorders need to have a sound understanding of ADHD to ensure accurate diagnosis and optimal targeting of evidence-based treatments. Misdiagnosis leads to patients being prescribed ineffective medications for symptoms which are secondary to ADHD. For example, affective symptoms such as emotional instability, which commonly co-occur with ADHD and show good response to ADHD treatments [86], may be misattributed to mood or personality disorders; or mind wandering in ADHD [87] may be attributed to a primary anxiety or mood disorder. Untreated ADHD may also prevent a positive treatment response for other common mental health conditions (e.g. [88]).

More recently, a rise in rapid poor-quality assessments leading to the inappropriate diagnosis of ADHD and initiation of ADHD medication in adults has also been noted by some specialists. This is clearly a potential problem that may increase in the future, requiring assessments to be carried out by individuals with sufficient training and expertise in the diagnosis and management of ADHD.

### The role of primary care

The skills required to appropriately diagnose and treat ADHD may also be provided by appropriately trained primary care clinicians with a background and training in the diagnosis and treatment of common mental health disorders. Although currently rare, there are examples of primary care clinicians with the required competencies to diagnose and treat ADHD. Decisions to refer to more specialist services may depend on a combination of the complexity of the clinical case, the competency of the clinician, and (unfortunately) the existence of somewhere to refer them to.

### Assessing ADHD

Straightforward cases can be diagnosed based on a clear account of current ADHD symptoms and impairments, and a history of persistent trait-like course of at least some of the symptoms from before age 12. In such cases, a diagnostic assessment may be feasible within 2 hours. Additional time is then usually required for initial psychoeducation and discussion of a treatment plan. The diagnostic assessment requires at a minimum a focused assessment at interview of ADHD symptoms and impairments, informant account from childhood where feasible, an account of the course and impact of ADHD symptoms from childhood, and assessment of common comorbidities.

In other more complex or subtle cases, increased investment in time or expertise may be needed to gather more information, sometimes over more than one appointment. Like other common mental health conditions, ADHD is a heterogeneous disorder, with variations in severity of symptoms and impairments, age of onset, presentation, multi-comorbidity, and substance use and abuse.

### Treating ADHD

Recommended treatments include psychoeducation, psychosocial support, environmental modifications, and medication. NICE recommend that psychological treatments such as cognitive behavioral therapy are generally reserved for those who do not adequately respond to medical treatment or choose not to take medication. This guideline is based on the conclusion that the weight of evidence indicates that core symptoms of the disorder are only effectively treated with medication [89–92]. However, non-pharmacological approaches remain important for helping patients to better manage the symptoms and impairments of ADHD, as well as the treatment of common comorbidities. Pharmacological treatment with concomitant psychoeducation is therefore recommended by all national and international guidelines as the mainstay of treatment for ADHD. Psychological treatments remain particularly important for comorbid problems such as anxiety, depression, personality disorder and substance misuse.

Medications for ADHD are relatively well tolerated, safe and effective [92]. Treatment initiation and monitoring requires the prescriber to understand the properties of the medications, their possible interactions with other conditions and drugs, and potential adverse effects. While in most cases initiation of medication for ADHD is a rapid and straightforward process, in other cases more time or skill is required. Those engaged in initiating medications for ADHD should be aware of the impact on symptoms and function in daily life and be able to provide guidance on coping with symptoms and impairment.

Access to ADHD-specific psychological interventions is very limited in NHS services and many other regions and countries. There is a clear need to incorporate training on adult ADHD into clinical psychology, nurse mental health and occupational health training. In the UK, psychological support for ADHD should be accessible through the Improving Access to Psychological Therapies (IAPT) program, which is a program for the rapid delivery of evidence based psychological treatments for common mental health disorders.

### Organisation and resourcing of services

NICE guidance advises that the exact balance between primary and secondary care will vary depending on the circumstances of the person with ADHD, and the available primary and secondary care services [48]. This allows for significant variation in service delivery arrangements for adult ADHD. The 2018 CATCh-uS mapping study identified a wide range of NHS services delivering provision for adult ADHD. These included specialist dedicated ADHD services, neurodevelopmental services, generic adult mental health services (AMHS), learning disability services, drug and alcohol services and autism services. Survey respondents in CATCh-uS also highlighted that adult ADHD input was also sometimes accessed via generic Child and Adolescent Mental Health Services (CAMHS), and specialist children’s ADHD services, particularly with regard to the continuation of treatment started during childhood and adolescence [51, 65].

**Fig. 2:**
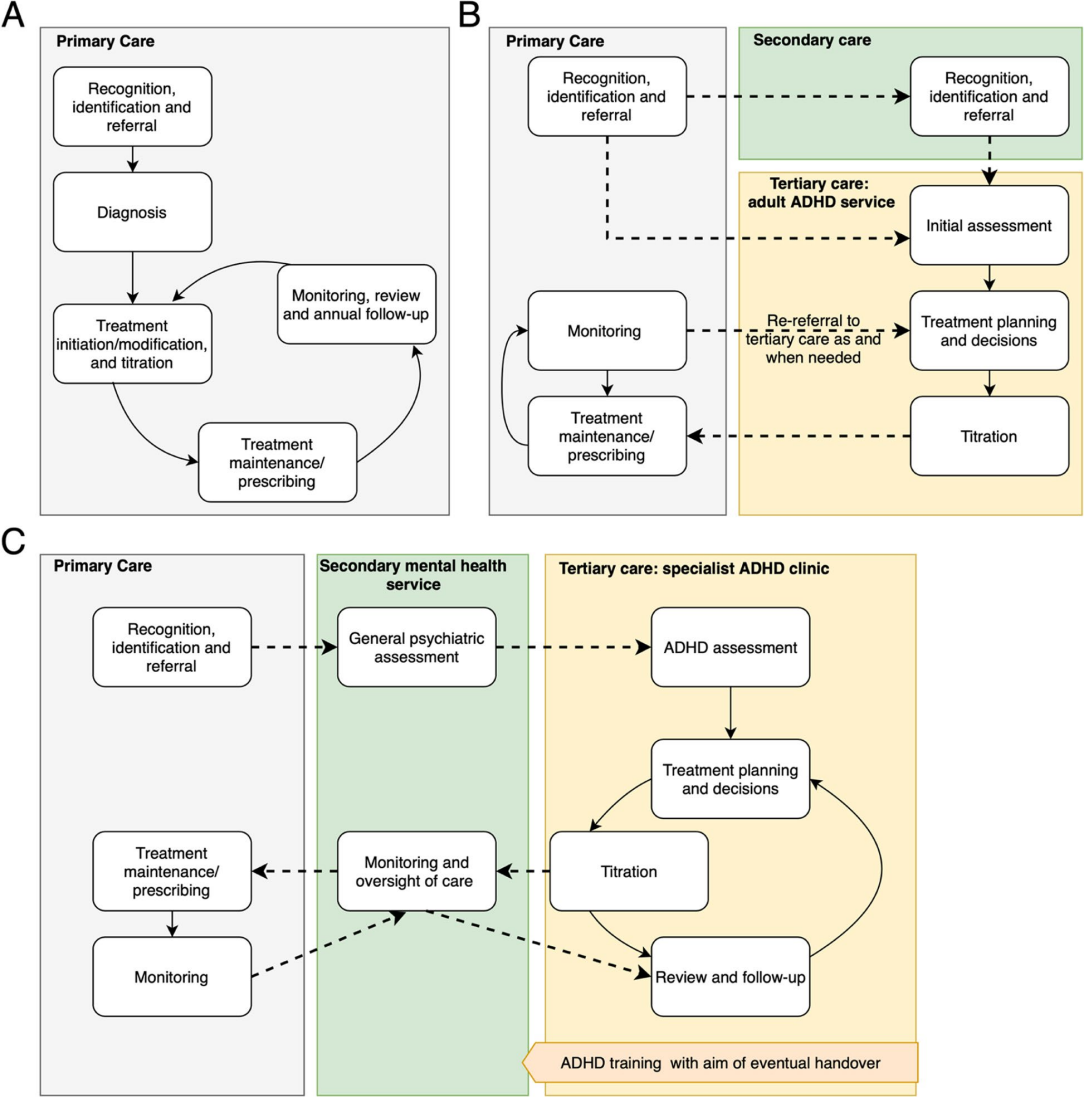
Different ADHD clinical care provision models in England. Dashed lines indicate key areas of communication between general and specialist healthcare providers. Case **A** Primary care model in North Bristol, delivering mental healthcare in GP surgery by specialist mental health nurse alongside other common mental health problems (depression, anxiety disorders). Dealing with < 100 referrals at date of consensus meeting. Issues arising: transfer of care over to other primary care services where ADHD diagnoses are not recognised. Case **B** Hybrid service in the Wirral, taking referrals both from primary and secondary care. Some transfer of specialism into primary care with the development of GP hubs who complete annual reviews and freeing up specialist time for new assessments and more complex cases. This service currently manages approximately 500 referrals per year. Issues arising: sudden restriction of medication prescribing in primary care through prescribing formularies, financial limitations, and concerns about funding diversion from secondary into primary care. Case **C** Tertiary ‘light’ service model in Leicester, working closely with secondary service and providing training with long-term aim to transfer care of ADHD into secondary healthcare. Well supported by healthcare commissioners and currently dealing with over 1000 referrals per year. Issues arising: high caseload in secondary care restricts capacity to take on ADHD cases, even for secondary care clinicians with adequate training. The number of required annual reviews has built up over time to the point where tertiary care is struggling to manage caseload

As well as variation in the types of services delivering ADHD provision, there is significant variation in how these services are organised. Examples of some of the different models of service delivery currently in use are shown in Fig. 2. An increasing number of mental health teams are incorporating ADHD diagnosis and treatment into generic AMHS as part of community mental health teams however, there is usually more of a hybrid approach (Fig. 2B). Although rare, there are some examples of specialist GPs and nurse practitioners/prescribers successfully diagnosing and treating adults with ADHD within primary care. More commonly, a mental health professional is embedded within primary care (Fig. 2A). In some cases, there are specialist ADHD services, segregated from both primary and secondary mental health services (Fig. 2C). Some pharmacists are also engaged in follow-up and monitoring of pharmacological treatments.

The examples provided here are by no means exhaustive but provide an indication of the different degrees of integration or segregation of ADHD across the healthcare system. Furthermore, they identify areas of vulnerability within these models, where lines of communication and cooperation between separate services can break down, and demand can start to outstrip service resources.

**Fig. 3:**
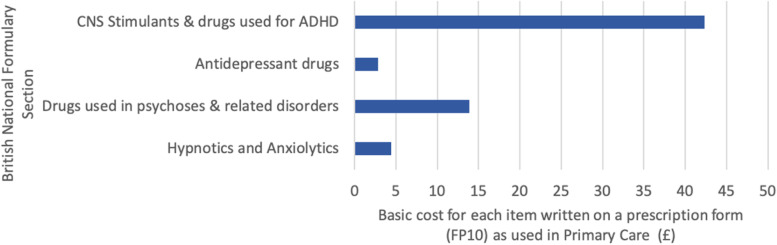
Basic net ingredient cost in 2018 for each item as listed on a prescription form in primary care (known as an FP10), categorised by British National Formulary (BNF) Section. Each single item written on the form is counted as a prescription item. For ADHD medications, one packet is usually a month’s supply in keeping with the recommendation to prescribe controlled medications for no more than 30 days. Data taken from ‘Prescription Cost Analysis’ datasheet on NHS Digital Website [94]

Within the NHS in England, variation in ADHD service provision is influenced by regional differences in regulation and funding allocation. Local prescribing formularies, put together by a local committee of health professionals under each healthcare commissioning body [93] can prohibit effective shared care by barring the use of certain ADHD medications in primary care at a regional level, effectively limiting primary care involvement in prescribing. Local medication formularies take into consideration the cost effectiveness and resource impact of each medication [93], leading to inconsistencies from one healthcare commissioning body to the next. Medications for ADHD are costly compared with most other common mental health medications (Fig. 3, [94]). Prejudice surrounding ADHD may impact local decision making.

In England, many other issues in delivery of medical treatments for ADHD arise, which likely play a role in many other regions and countries. Compliance with local formularies may be enforced on a primary care service level by Medicines Management Teams. They can hold back funding for primary care services where they fail to adhere to local prescribing guidelines. In some areas, prohibitions from local prescribing formularies have been imposed suddenly, leaving primary care practitioners exposed to challenge when attempting to maintain treatment for their patients. The national contract states that primary care prescribers must prescribe what they think is the best treatment within the limits of their competence, however the local healthcare commissioning groups may advise them not to prescribe some drugs for ADHD. The General Medical Council says that doctors should prescribe a licenced drug rather than an unlicensed equivalent where possible. There are clearly competing and overlapping agendas, and a general lack of clarity in this area in England, which are likely reflected elsewhere.

Resource limitations can reduce capacity for primary services to support shared care in ADHD treatment monitoring and prescribing. Even when formularies allow prescribing and primary care representatives agree that pathways are clinically appropriate, some practices argue they lack capacity to accept shared care arrangements without additional funding. Additional training also requires funding.

Primary care practices typically have an ‘indicative budget’ and ‘medicines management’ is typically part of a local ‘Prescribing Incentive Scheme’. Each regional healthcare commissioning body decides how to run its own budgets, and this can mean regionally divergent rules on what primary care practices must do to receive incentives. This usually includes some ‘quality’ issues such as audits to show the appropriateness of prescribing (i.e., not just cost), but may also include keeping within the indicative budget. An average sized practice which fails to meet requirements for a local medicine management scheme, may miss out on funding in the order of several thousand pounds. Further, if the regional healthcare commissioning body exceeded its budget, they are required to make cuts or claw back funds from other areas of healthcare. This is a complex process which varies between regions.

In regions of England without adult ADHD services, individuals should by right, be able to access these services elsewhere. A patient’s legal “right to choose” the providers of their care was introduced in 2014 and updated in 2018 [95]. With the increasing provision of ADHD diagnostic assessments by on-line providers with NHS contracts, it is possible that patients will exercise their choice to obtain assessments more quickly or conveniently via these non-traditional routes. While the expansion in capacity for ADHD services should be encouraged, there is a risk of diverting funding from existing local services. From the healthcare commissioning perspective, the ‘Choice’ agenda can undermine regional commissioning and resource allocation intentions, which may prompt healthcare commissioners to manage demand through referral management schemes.

### Developing the role of primary care in adult ADHD

#### The impact of ADHD in primary care

ADHD in adults often presents within primary care with complaints of poor functioning or performance, educational or occupational failure, emotional instability, anxious worrying, mood symptoms or sleep problems. ADHD is also associated with increased rates of general health conditions such as obesity, type II diabetes, asthma, hypercholesterolaemia, hypertension and smoking and its consequences [25, 96–98]. Thus, many of the presenting complaints and impairments of ADHD overlap with common health conditions that are typically managed within primary care.

There is increasing evidence that improved management of ADHD can help a range of co-occurring health problems, in addition to reducing the core symptoms and impairments of ADHD. For example, ADHD is associated with treatment resistance in comorbid depression [99] which may be reduced by regular treatment for ADHD [88]. Rates of depression, mania, emotional dysregulation and substance abuse may all reduce during treatment of ADHD with stimulant medication [30, 100]. Common physical health problems are also increased in adults with ADHD, perhaps due to poor self-regulation of behaviour and lifestyle factors. Good management of ADHD may help to decrease risk factors for health problems such as smoking and obesity, as well as enhance the self-monitoring and management of chronic conditions such as diabetes and hypertension.

Pharmacotherapy for ADHD does appear to be a protective factor for obesity [101], communicable diseases [40, 41] and epileptic seizures [39]. Improving access to treatment and support for individuals with ADHD within primary care is therefore likely to have benefits for treatment efficacy across other physical and mental health conditions, in addition to common mental health complaints, which are typically managed within primary care.

Consistent with these findings is the evidence that treating ADHD in adults reduces health care and other public services use costs further down the line [31, 32, 102]. The available evidence makes a strong case for improving capacity for treating ADHD based on a ‘spend-to-save’ logic, since the high prevalence of adult ADHD combined with the burden of comorbidity means that failure to meet the health needs of this group will incur societal costs which exceed those of an effective medical service. At present there is no clear evidence regarding the rates of unrecognised ADHD in primary care, and which conditions patients with undiagnosed ADHD are receiving treatment for, but this area warrants further research. The opinion of the experts at the meeting was that ADHD would be seen in around 10–20% of those attending primary care services with chronic mental health problems, and a higher-than-expected rate in those with common physical health disorders.

Improving capacity for managing ADHD by primary care clinicians who are already tasked with managing a range of health conditions commonly comorbid with ADHD, could help to improve health outcomes more broadly and result in longer-term savings. Arguably, effective management of ADHD could reduce overall work volume due to the positive impact of other mental and physical health issues, resulting in better utilisation of GP resources.

#### Shared care for treatment maintenance and monitoring – the status quo

As discussed above, NICE guidance recommends that ADHD can be managed and monitored jointly between specialists and primary healthcare, under shared care protocols [48]. As recommended by NICE, primary healthcare providers should contribute to shared care for ADHD by taking over routine prescribing and physical monitoring (weight, blood pressure and heart rate, minor adverse effects), after patients have been stabilised on pharmacotherapy. Primary care can also support adults with ADHD by referral to psychological services for non-pharmacological support. Transfer of routine follow-up of patients with ADHD to primary care can help to free up capacity within secondary or tertiary mental health services, allowing them to take on new referrals and manage more complicated cases. In turn, secondary or tertiary services should support shared care arrangements, facilitate appropriate training, and provide open lines of communication and advice on patient care.

Sometimes shared care arrangements are drawn up by secondary or tertiary services, or healthcare commissioners, without sufficient primary care input. Some primary care practitioners are concerned about this shift in workload from secondary to primary care without additional or sufficient resources [103]. Funding for primary care in England decreased by 6% in real terms, from 2005 to 2006 to 2013–2014, with a simultaneous estimated increase of 16% in overall workload. This compares with an increase in real-term secondary care funding of 2% per annum [104], although mental health services may not have shared in this growth. When considering the shifting of clinical responsibilities between healthcare sectors, additional resources should be provided as required.

Not all primary healthcare practitioners support shared care protocols, and in some areas primary care will not take up the responsibility for physical monitoring and continued prescribing. Sometimes GPs will point out this not part of their contract and they are not funded for this work. Primary care practitioners may also have valid concerns about taking on clinical responsibility for an unfamiliar treatment and disorder [105], particularly in the context of insufficient communication with ADHD service providers, insufficient training, lack of clear protocols for shared care or for monitoring treatment, or concerns around inadequacy of treatment monitoring [79, 106, 107]. In other instances, barriers to shared care include stigma and concerns around the legitimacy of an ADHD diagnosis [13, 54, 77, 108, 109]. In many instances, problems with shared care could be addressed through improved communication, training, and education and this should be provided and appropriately funded (including backfill for covering study leave).

Routine follow-up of ADHD patients is not simply a matter of monitoring physiological parameters, but also of understanding how the condition is currently impacting on the person’s life, including the impact of treatment (or non-treatment) on any comorbidity, and the provision of psychosocial support where required. According to NICE, a formal review needs to be carried out at least annually, by a clinician with a good understanding of this condition. Adequate training and support for primary care practitioners is therefore needed to support routine follow-up, with access to specialist ADHD services for ongoing advice as required.

#### The challenge of increasing demand

**Fig. 4:**
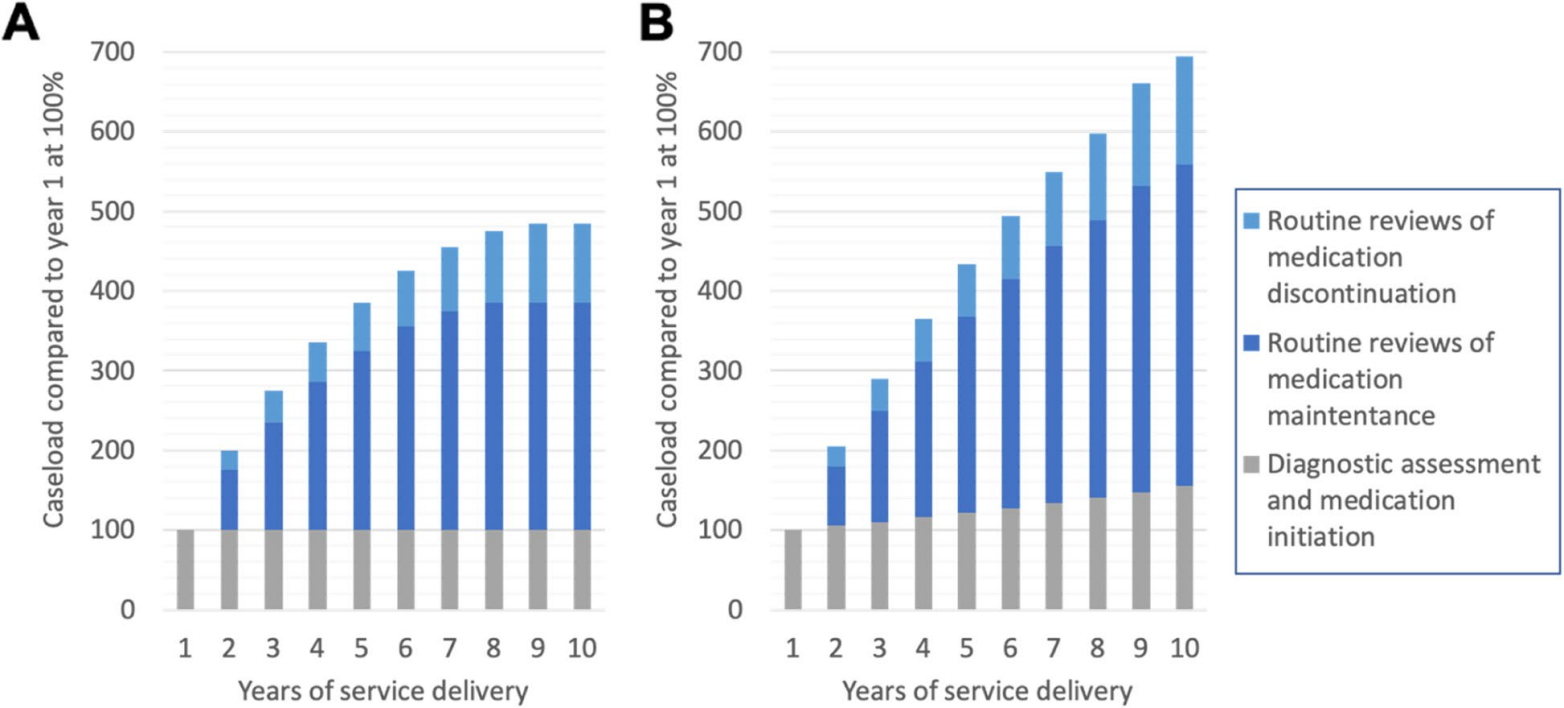
Simplified schema of hypothetical clinic caseload in the first 10 years of ADHD service delivery with no limit to growth in provision of funding or staff resources. With reference to year 1 at 100%, and assuming the following rates of medication discontinuation: 25% decrease in medication one year after diagnosis and a 10% yearly drop after this [110], and that ADHD patients are only seen in secondary services for routine follow-up in the first year after medication discontinuation. **A** clinic caseload in the context of stable referral rates, **B** clinic caseload in the context of a 5% yearly increase in referral/diagnosis/treatment rates. There will be other complicating factors beyond the scope of this model, such as migration in/out of catchment, increasing awareness in population over time, potential for diagnostic thresholds to change with revisions to diagnostic criteria

Even in the context of shared care arrangements, demand for resources from specialist teams increase with every newly diagnosed patient. A large population study from Sweden found that following initiation of medication for ADHD, 75% of adults were still receiving medication after 1 year, 60% after 2 years, 50% after 3 years, and 42% after 4 years [110]. Assuming similar rates of medication discontinuation, a constant rate of referral, and that ADHD patients are only seen in specialist services for routine follow-up in the first year after discontinuation, ADHD clinics are likely to see their caseload double within the first year after opening, and treble after 4 years (see Fig. 4A for a simplified schema). With a modest increase in diagnosis and treatment rates at 5% annually, the caseload of ADHD clinics increases even more rapidly (Fig. 4B).

Routine annual reviews of patients take up an increasing portion of finite clinical capacity. Over time, clinics with no growth in funding or staff resources see the service getting clogged up with routine annual reviews, constricting resources for not-yet diagnosed or treated patients. This issue, coupled with increasing rates of referral resulting from increasing public and professional awareness of the condition and the high prevalence of ADHD, have led to demand outpacing provision, and in turn to very long waiting lists.

### A potential solution: a hybrid primary-secondary specialist service

A hybrid specialist service, which transfers annual treatment reviews to qualified staff within primary care can help to reduce incremental resource demands on secondary health services. As described previously, most primary healthcare practitioners are well qualified to obtain the adequate training and supervision required for long term monitoring of ADHD. Annual treatment review for straightforward cases within primary care would allow secondary care to focus more on new patients and those with a more complex clinical presentation. The annual reviews could be completed by primary care clinicians with specialism in ADHD, such as specialist mental health nurses, psychologists, pharmacists, or general practitioners with a special interest in ADHD (see Fig. 2B for an example of this service delivery model). This transfer of responsibility should occur alongside a reallocation of resources. The problem in the UK is that Primary care and Mental Health services are already felt to be severely underfunded, so stakeholders may reasonably argue that they should neither give up any existing funding nor take on additional work. With respect to optimising access to ADHD services, and reducing waiting times, this model is preferable to the standard shared care arrangements described above.

### Overcoming the funding stalemate

Political motivation will be needed to improve services for ADHD by mandating and funding care for ADHD across primary, secondary, and tertiary services.

Funding arrangements to support resource allocation between services are not established for ADHD. For example, shared care arrangements are not delineated in most primary care contracts. The General Medical Service (GMS) contract with primary care is to assess and treat or refer people who may be unwell. Chronic disease monitoring and preventive care is part of the Quality and Outcomes Framework (QoF), but is optional, and currently attracts funding for provision of specific areas of care that do not include ADHD. Moreover, the QoF framework does not provide a catch-all for all follow-up and long-term monitoring requirements. Resource allocation for ADHD treatment monitoring could be provided by incorporating funding for care of ADHD under the QoF in a similar way to funding for diabetic care. Similarly, it would be possible to set up a “Direct Enhanced Service” as an addition to the core GMS contract, with its own national funding.

Currently, if a healthcare commissioning group wishes to fund care for ADHD locally, it is possible to set up a “Local Incentive Scheme” (also known as a “Local Enhanced Service”) to fund primary care to support monitoring as part of shared care. There are issues surrounding equity of access unless all practices sign up to provide this service, and the healthcare commissioners must decide where else to divest to fund the scheme.

The precise model or contractual framework may be much less important than the political will to address the needs of the population with ADHD. The will to improve provision for mental health more generally is confirmed, at least on paper. The ‘Community Mental Health Framework for Adults and Older Adults’ 2019 [111] mentions ‘neurodevelopmental disorders’ as a comorbidity contributing to ‘complexity’ however it does not mention ADHD specifically, whereas many other conditions are listed. However, the aims of the framework are promising, and include improved support for the primary care workforce around mental health, through an increase in skills and knowledge, rapid access to expert mental health clinical advice, and fewer rejections of referrals.

### New horizons - the mainstreaming of adult ADHD

Further mainstreaming of adult ADHD by incorporating it more into general adult mental health services, is likely to have an important impact on enhancing access to diagnosis and treatment. As discussed, a key initial change to service organisation would see the transfer of routine annual medication monitoring to primary care for patients with a positive and stable response to their treatment regimen. Referral for more specialist input would be only where there is a specific issue or area of unmet need, including the delivery of psychosocial interventions. A related model could see referral to, for example, nurse led teams that sit between primary and secondary care, for long term monitoring of the disorder, again with onward referral as required. Nurse and psychology led services may be better at providing ongoing psychoeducation and psychosocial support in addition to monitoring of medication,

Formal training and accreditation for specialism in ADHD diagnosis and treatment could be arranged through primary care training hubs, or with support from professional membership bodies, such as the Royal College of GPs or the Royal College of Psychiatrists in the UK. Initially this may be particularly important for primary care staff, for whom involvement in adult ADHD management is currently less common than for secondary or tertiary care staff.

In the longer-term, a care pathway modelled on the NICE stepped care model for anxiety disorders and depression [112, 113] could be effective for ADHD, bringing ADHD management in line with other common mental health conditions. Under this model, with the necessary training, primary care ADHD specialists (GPs or embedded mental health practitioners) may be able to take over treatment initiation and titration for patients with more straightforward clinical presentations. More subtle or complex presentations are referred to generic mental health services, or specialist services. It is not realistic for every primary care service to use this approach as many are not large enough to warrant in-house expertise. A comprehensive assessment is likely to take 2 h or more of a clinician’s time, and would not be not possible with the constraints of typical primary care contacts [114]. However, primary care may be ideally suited to conduct assessments if they have known the patient over an extended period, can gather information with the assistance of multi-disciplinary team, and include a clinician with specialism in ADHD.

Primary care ADHD services could also be incorporated within Primary Care Networks (PCNs). These are part of the NHS Long Term Plan published in 2019 [115], which describes the planned development of PCNs which incorporate neighbouring GP practices and a range of services (community, mental health, social care, pharmacy and hospitals and voluntary services) in local areas which work and are funded together, typically covering between 30,000–50,000 patients. Collaboration between practices and services allows for larger multidisciplinary teams to cover the broader area, and potentially provide better access to specialist health professionals and services which are shared across the local area. PCNs are charged with developing these local and flexible health solutions. Using a PCN approach it would be possible to set up primary care hubs for ADHD which can serve local populations. The development of PCNs supporting better management and treatment of adult ADHD in the context of more general mental health care provision can be readily justified, given the personal and societal costs of adult ADHD, the demonstrated benefits of treatment, and the current bottlenecks in service delivery.

Increasing knowledge about ADHD within multidisciplinary primary care practitioners would have a powerful role in improving ADHD provision. Teams can work together to reach consensus when the threshold of complexity is reached. Accessibility and clear lines of communication to ADHD specialists is needed.

An important component of primary care mental health are psychological services. In England, so called IAPT Services (Improving Access to Psychological Therapies) typically provide psychological therapies for adults with anxiety disorders and depression [116], but currently do not focus on ADHD. IAPT interventions for ADHD should now be included given the high frequency of comorbidity, in addition to the need to teach robust coping skills for ongoing ADHD symptoms and impairments. A trial program in Cambridge (England) that has integrated the medical care for ADHD with psychological support from the primary care psychological (IAPT) service was reported to be working well and the consensus group’s view was that this should be extended to other areas. This highlights the important role that primary care can take in delivering psychological support for the management of ADHD.

## Discussion

### Overall conclusions

Whilst the last two decades have seen a stepped change and increase in the provision of adult ADHD clinical services in the UK and elsewhere, demand currently outstrips provision by a long way in many regions and countries. Growing awareness of the condition in the public and clinical community has led to increased referrals for adult ADHD. Trends showing yearly increases in ADHD treatment in the population [49], coupled with evidence that rates of diagnosis and treatment are far below prevalence of ADHD in the community, suggest that demand will further increase in years to come. ADHD is a common mental health condition [5], but at present is treated as more of a niche problem, with diagnosis, treatment initiation and monitoring frequently constrained to scarce specialist services with limited capacity. The result of a high prevalence in combination with relative paucity of services, is that many people with ADHD experience limited access to care, and extremely long waiting times before they can access the care they need.

People with ADHD are vulnerable to other concurrent mental health problems, alcohol and drug use problems, educational and occupational impairments, accidents and injuries, communicable diseases, and involvement with the criminal justice system [15, 17–20, 22, 25–27, 40, 41, 117]. ADHD is also associated with increased levels of homelessness in adulthood [23]. Impairments and psychosocial burden are likely to develop and accumulate over time, and engaging with ADHD treatment is expected to reduce longer-term risks and improve outcomes [29, 30, 100]. Delays in treatment will reduce economic productivity, and increase burden on health, social care, criminal justice, and state benefits [31, 32, 118]. It is therefore in the interest of the broader health and social care sector that individuals with ADHD are provided with adequate and timely support and treatment.

At the heart of the problem, we find a trio of interrelated constraints. The first pivots on the interpretation and understanding of ‘ADHD specialists’, who are responsible for delivering a range of services to support adults with ADHD [48]. Whilst NICE guidelines give broad brushstrokes on the clinical qualifications and competencies required, they lack clarity and specificity. This leaves room for unhelpful debate regarding which service providers should take ultimate responsibility for delivering adult ADHD services, or the assumption that ‘specialist’ simply refers to specially qualified secondary services. Since adult ADHD is currently not incorporated in generic adult medical or psychiatric training, there are no de-facto ADHD specialists based on clinical qualifications alone. Rather, ADHD specialism is acquired through additional professional training and development.

In the current paper we review NICE guidelines and conclude that adult ADHD specialist support can be delivered at primary, secondary, or tertiary level, assuming appropriate levels of professional training and supervision. Adopting a more inclusive view of ‘ADHD specialists’ can help to improve access to treatment for patients by broadening sources of clinical expertise.

The second constraint is organisational. Where there is over-reliance on the limited capacity secondary and specialist services, these rapidly become overwhelmed when treating this common and often chronic condition resulting in bottlenecks. Transfer of routine clinical care (routine prescribing and physical monitoring) to primary care providers through shared care protocols can help to alleviate some of this strain on capacity and should occur as standard clinical practice. However, even in the context of effective shared care protocols, ADHD specialist clinics with no growth in funding or staff resources often become clogged up with routine annual reviews and become unable to accommodate new referrals in a timely fashion.

The third constraint is financial. Different commissioning arrangements for primary and secondary healthcare can lead to tensions regarding transfer of routine care between services, particularly for patients with ADHD in whom pharmacological treatments are often provided long-term and are more expensive than for other mental health conditions. Limits to budgets and competing demands from different parts of the healthcare sector results in a reluctance to take on additional work, with ADHD often falling between services. There is no cost neutral solution which will solve the capacity issue and therefore models should not be built around cost-saving or cost-shifting, but rather a longer-term perspective built around the question ‘what does good care look like?’

Although the focus on services in England is a potential limitation of this paper, the issues raised are widely experienced and are of general relevance to service development for ADHD in many other regions and countries.

## Recommendations

Based on these discussions the group made a set of recommendations for the future development of primary care services for adults with ADHD:

1. Mainstreaming straightforward cases of ADHD into primary care could enhance access to diagnosis and treatment for this common condition, improve the health and wellbeing of adults with ADHD, and reduce the burden of co-occurring problems and conditions on the broader health system. These changes to ADHD service delivery should be supported by patient consultation, to examine their perspectives on service models and preferences as to where and how their care is managed [119, 120].
2. Changes to operational models could be supported by formal training and accreditation for specialism in adult ADHD. An initial change to service organisation would see the transfer of routine annual medication monitoring duties (the ‘annual review’) to primary care for patients with a positive and stable response to their treatment regimen, and referral back to specialist services only in instances when treatment requires attention or modification.
3. ADHD Specialism embedded within primary care will also help to facilitate transition from child to adult services, by maintaining ADHD management until adult services are available.
4. For students with ADHD, student disability teams and trained specialist assessors who complete diagnostic assessments for ADHD should collaborate with clinical services for ADHD. Their assessments can then form part of the medical assessment through appropriate collaboration with either a student secondary care mental health service or trained health care professionals within primary care. An ADHD service embedded within primary care, especially at surgeries located within university health centres, can offer a cost-efficient pathway for university students with ADHD.
5. In the longer-term, ADHD treatment and management should be brought in line with care pathways for other common mental health disorders, where primary care practitioners with expertise in ADHD take over treatment initiation and titration for patients with more straightforward clinical presentations. Referral to generic mental health or specialist services is indicated for more complex cases. The management of uncomplicated adult ADHD could follow the model of other common mental health problems, which has become core business of primary care and IAPT over the last 20 years.
6. Such developments would require greater resourcing for mental health within primary care. It is unrealistic to expect each primary care service to have an in-house ADHD specialist, but plans for more joined up and flexible provision and funding arrangements hold promise for improvement in the delivery of ADHD services. Collaborating practices, using a primary care network approach, can invest in developing shared ADHD specialist resources across a larger area which serve a larger portion of the population. In turn, with support from appropriately experienced and qualified primary care networks, individual primary healthcare providers may be better supported to take on routine treatment and follow-up assessments, and broader facets of ADHD management and treatment.

## Data Availability

The recording of the meeting was destroyed once they had been transcribed. An anonymised transcript is available from the corresponding author on reasonable request; but is not publicly available as consent was not obtained for public sharing.
